# Sifting fluctuation scattering from microtextured samples

**DOI:** 10.1107/S2052252522002044

**Published:** 2022-02-26

**Authors:** Antonio Cervellino

**Affiliations:** a Swiss Light Source, Paul Scherrer Institut, Forschungstrasse 111, 5232 Villigen Switzerland

**Keywords:** correlated fluctuations, dynamical studies, XFELs, X-ray free-electron lasers, fluctuation scattering, preferred orientation, pair-angle distribution functions, intensity correlations, crystalline domains

## Abstract

As the relationship of texture and microtexture to fluctuation X-ray scattering (FXS) has been clarified in detail, key progress is expected in the exploitation of FXS-based structural investigation of matter exhibiting complex order.

The paper by Binns and coworkers in this issue of **IUCrJ** (Binns *et al.*, 2022 ▸) contains a key finding for promoting the structural investigation of matter exhibiting complex order by fluctuation X-ray scattering methods (FXS). As a result of the deep and accurate theoretical analysis thereby exposed, substantiated by careful experiments, one of the strongest limits of the technique, its sensitivity to microstructure, has been overcome.

A brief outline is in order. It is nowadays assumed that by X-ray diffraction we can determine the atomic structure of a crystal. Much of the structural information is destroyed when we have a large number of isotropically and homogeneously oriented crystals instead of a single one (as in a powder). The directional averaging means that the diffraction pattern is substantially 1D. If sharp Bragg peaks are the dominant feature of the pattern, they can be lifted in 3D momentum space and the structure can be solved as well, albeit with more difficulty and some limitations. For a powder with particles that are not (or are imperfectly) crystalline, we can reliably recover only the scalar pair correlation or pair distribution function (PDF) from the scattered intensity, but no higher correlations. So a complex deduction game must be played to translate interatomic distances – directly gleamed as sharp peaks of the PDF – into 3D geometry. Kam (1977 ▸) thought to exploit the then novel 2D detectors and add fluctuation X-ray scattering (FXS) into the frame. FXS is a minor component of the scattering from an ideally isotropic powder, arising from zero-average fluctuations of the orientation distribution of the constituent particles. Separating FXS after subtracting the powder signal (the constant-momentum average of the pattern) was shown to yield higher-level information, namely the pair-angle distribution function (PADF) (Martin, 2017 ▸), that includes three- and four-body correlations with distances and angles (*cf.* Fig. 1 ▸).

Clearly, the exploitation of FXS to yield PADF brings us much closer to understanding structures of less-crystalline matter (*e.g.* liquid crystals and nanoparticles within or without self-organized supercrystals). The FXS signal is, however, weak and can only be extracted if we can subtract the baseline powder diffraction signal. This is straightforward with ideal powders.

Enter preferred orientation (texture) and the situation becomes much more complex. Basically, texture introduces an *a priori* unknown angular variation of the baseline powder signal, which is then added to the FXS signal but does not contain structural information, while easily strong enough to swamp the FXS signal. Texture has long since been the bane of powder crystallography; its effects can be dramatic in falsifying the Bragg intensities, and it also affects the PDF (Cervellino & Frison, 2020 ▸). Huge efforts have been devoted to understanding and characterizing texture (Roe, 1965 ▸; Bunge, 1987 ▸) in order to develop useful correction algorithms. Moreover, sample preparation methods have been developed to reduce *macro*texture – the texture that is determined mainly by sample preparation and containment, and whose description is unique for the whole sample. However, there is often also *micro*texture – preferred orientation on a local scale, much bigger than the powder particles but much finer than the sample dimension. This is locally variable, depending on local conditions, so no sample-wide correction is possible. It is also typically not so strong as macrotexture can be; however, it can still easily be stronger than FXS. Not always of course, otherwise we would not have had a successful history of FXS experiments. Microtexture adds this margin of unpredictability that strongly dampens expectations of success in an experimental campaign based on FXS.

The importance of Binns and coworkers’ paper (Binns *et al.*, 2022 ▸) is that it provides an accurate analysis of the effects of microtexture on FXS. It also presents an important result in terms of the intensity ratio of the microtexture signal to the FXS one. This ratio scales linearly with the number of illuminated grains, *i.e.* with the illuminated sample volume. This means that thinner beams will give more relevance to the FXS signal. This is especially good news because it leverages the great developments of the last decade (Shin, 2021 ▸) at synchrotron and XFEL sources (but also in laboratory instruments) in terms of brilliance. Between many other positive effects, a high brilliance facilitates the production of stable microfocused beams, down to well below the micrometre range. Increasing source brilliance means that very thin beams are still powerful enough to give a clear FXS signal within the necessarily short acquisition time while depressing the microtexture signal into irrelevance by their tiny size. In Binns *et al.* (2022 ▸) a brilliant experimental test is shown to highlight this feature. An experiment with a large beam (a few thousands of square micrometres) shows only the microtexture signal, while another experiment with a thin beam (a couple of square micrometres) on the same substance (a well reproducible liquid crystal mesophase) clearly showed the FXS signal.

And this is just the beginning, of course. A clear linear scaling behaviour of the FXS to texture ratio will open the door to more complex but still easily feasible experimental setups. These may involve rapidly variable beam spot size coupled with fast area detectors and principal component analysis. And FXS could be poised to become a mainstream method, finally leaving its obscure niche.

## Figures and Tables

**Figure 1:**
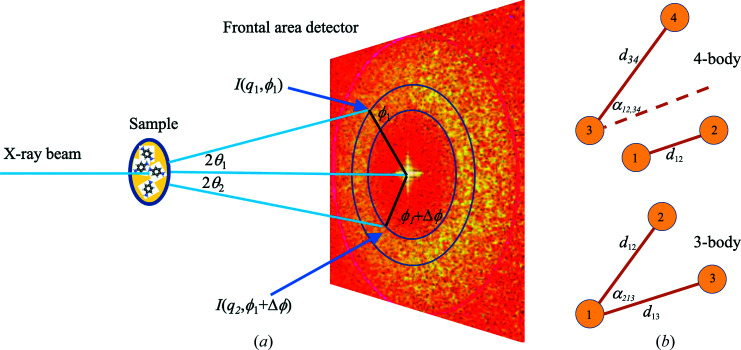
(*a*) The power of FXS is that, summing the products of intensities with two different *q* values and with constant angular separation 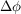 , one obtains not only the interatomic vector distance lengths as from standard 1D PDF, but also (*b*) the angle between two interatomic vectors. These can share a common atom (three-body correlation) or not (four-body correlation).
